# Therapeutic potential of *Ficus benghalensis* in thromboembolic disorders

**DOI:** 10.1016/j.jaim.2024.100929

**Published:** 2024-08-05

**Authors:** Anil Kumar Sahu, Drishya Dinesh, Vipin Kumar Verma, Vaishali Prajapati, Jagriti Bhatia, Dharamvir Singh Arya

**Affiliations:** Cardiovascular Research Laboratory, Department of Pharmacology, All India Institute of Medical Sciences, New Delhi, 110029, India

**Keywords:** *Ficus benghalensis* L., Thromboembolism, Anticoagulant, Anti-thrombotic, Arterial thrombosis

## Abstract

**Objective:**

*Ficus benghalensis* L. (*FB*) is a popular plant described in the Indian system of medicine. Traditionally, it is indicated in the treatment of diseases like diabetes mellitus, dysentery, leucorrhoea, menorrhagia, skin disease, rheumatism, inflammatory diseases, blood disorders. This paper accentuates the anti-thrombotic action of *FB* based on the properties like anti-coagulant, platelet-antiaggregatory, anti-atherogenic hypotensive, hypolipidemic, anti-oxidant, anti-inflammatory and immunomodulatory.

**Methods:**

All the available data pertaining to *FB* has been searched in the scientific databases, including PubMed, Google Scholar, ScienceDirect and Scopus.

**Results:**

*FB* is a rich lode of organic compounds such as phenols, flavonoids, alkaloids, tannins, terpenoids and steroids. The various studies show that these phytochemical constituents exhibit wide range of anti-thrombotic actions such as anticoagulant, platelet *anti*-aggregatory, anti-atherogenic, hypolipidemic, hypotensive, anti-inflammatory, and antioxidant.

**Conclusion:**

Various studies (*in vitro* and *in vivo*) confirm the potential anti-thrombotic benefit of *FB* due to the presence of chemical structures that have proven to be effective in thromboembolic conditions. These evidences may benefit in new drug development to treat varied thromboembolic conditions which will not only be cost effective but may allay the fear of side effects.

## 1 Introduction

Cardiovascular diseases contribute to 32% of annual deaths worldwide [1] and distinctively, thromboembolic conditions account for 1 in 4 deaths. Estimates for the global incidence rate (IR) confirms 114.3 ischemic stroke, 139.3 for myocardial infarction, 1518.7 for ischemic heart disease, 77.5 for atrial fibrillation in males and 59.5 in females, and 115 to 269 for venous thromboembolism per 100000 population [2].

Thrombosis is the formation of a blood clot in the blood vessels, limiting the natural flow of blood and resulting in clinical sequelae. Embolus is a dislodged intravascular mass that can be solid, liquid or gas that travels with the blood from its point of origin to a distant site, leading to infarction or tissue dysfunction. The majority of the emboli are formed from dislodged thrombi; hence, it is known as thromboembolism [3]. Thromboembolic conditions can be classified into two categories: arterial thromboembolism (ATE), which majorly encompasses ischemic heart disease, chronic peripheral arterial disease, and ischemic stroke, and venous thromboembolism (VTE), including pulmonary embolism and deep vein thrombosis. The etiology of thromboembolism is multifactorial. Obesity, progressing age, high blood pressure, diabetes, high cholesterol level, sedentary lifestyle, cigarette smoking, mitral stenosis, and atrial fibrillation are the primary risk factors for ATE, whereas chronic illness, bed confinement, trauma, and oestrogen-based therapy are the major risk factors for VTE [4].

Thrombus formation and propagation depends on the damage to the vascular endothelial cells, the increase in blood coagulability, and change in blood flow. Arterial thrombosis mostly begins with the deposition of lipid plaques in the lumen of the artery, evoking chronic inflammatory cells and activating the platelets. These fatty plaques then evolve into fibrous plaques, which could disrupt into unstable atherosclerotic plaques, which in turn release additional pro-coagulating factors that activates the platelets, causing adhesion and accumulation, leading to clot formation [5].

Drugs play a crucial role in the prevention and treatment of thromboembolism. Anticoagulant medications that target pro-coagulant factors are the cornerstone to treat venous thromboembolism, whereas antiplatelet medications used as monotherapy or dual-antiplatelet therapy are typically used to treat arterial thrombosis. Anti platelet drugs namely COX inhibitors, ADP receptor antagonists, Protease-activated-receptor-1 inhibitors, and αIIbβ3-Integrin inhibitors, as well as anticoagulant agents including vitamin K antagonist, un-fractioned heparins, Xa factor inhibitors, thrombin inhibitors, and coumarin derivatives are the current standard care for the treatment [6]. However, anticoagulants are limited by certain factors such as unpredictability regarding estimation of drug dosage, safe drug levels for major surgery, management of haemorrhage and renal dependence as well as multi dose plans [7].

The Indian subcontinent is a rich source of medicinal plants that have been used in health care since ancient time. More than 70% of the Indian population still uses traditional medicine for a variety of reasons, including sociocultural factors; perceived safety of herbal drugs; easy availability; rising costs of modern drugs; subpar healthcare facilities; and adverse effects of modern drugs and non-compliance. Also, the growing importance of medicinal plants can be appreciated from various economic vantage points as well. The Indian Banyan Tree, known as “Bargad” in Hindi, is a large perineal tree indigenous to the Indian subcontinent. It is spread all across the country and is known as *Ficus benghalensis* (FB). Traditionally, each part of the plant is found to have various therapeutic uses with vast commercial potential. Phytochemical analysis of various extracts of plant parts revealed the presence of amino acids, vitamins, phenols, terpenoids, phytosterols, flavonoids, alkaloids, anthraquinones, as well as cardiac glycosides [[8], [9], [10]]. Although not much description is available in the ancient Ayurvedic literature narrating its use as anti-thromboembolic agent, however, pharmacological properties such as *in-vitro* anticoagulant property, *in-vitro* platelet *anti*-aggregatory effect, *in-vivo* blood pressure lowering effect, direct antiatherogenic effect, and hypolipidemic effect reported in various studies reflect its anti-thromboembolic activity [[11], [12], [13], [14], [15]]. In addition to this, properties including antioxidant effect, anti-inflammatory effect and free radical scavenging activity, have been delineated in several studies [16,17]. Hence, in view of the above-mentioned favourable pharmacological profile of *FB*, the present review focuses on exploring the prospect of its rational use for treating various thromboembolic conditions.

## 2 Methodology

Database searches using Google Scholar, PubMed, and Science Direct were conducted until 15th May 2023 to include up to date documented information in the present review article. The search was limited to English language papers. For data mining, the following MESH words were used in the databases mentioned above: *FB* traditional use, *FB* ethnomedicinal uses, *FB* phytochemical constituent, *FB* pharmacological studies, *FB* Phenolic, Terpenoids, Tannins, Glycosides, phytosterols compounds, *FB* pharmacognosy, *FB in vitro* study, *FB* in-vivo study, *FB* antioxidant, *FB* anti-inflammatory *anti*-aggregatory/antiplatelet, *FB* anti-atherogenic, *FB* hypolipidemic, *FB* hypocholesteraemia, *FB* anticoagulant, *FB* hypotensive, *FB* toxicity studies. In almost all cases, the original articles were obtained and the relevant data was extracted. The chemical structures were made using the ChemDraw software.

## 3 Ethnomedicinal consideration

*FB*, popularly known as *Nyagrodha* or *Vatta* in Ayurveda, is one of the most glorified sacred trees with an immense ethnobotanical history. It possesses vast medicinal properties and is an important ingredient in many Ayurvedic formulations. Traditionally, different plant parts have been used in many disease conditions like diabetes mellitus, dysentery, diarrhea, leucorrhoea, menorrhagia, skin disease, rheumatism, inflammatory diseases, blood disorders etc. (Table 1) [11,15,[18], [19], [20]].

**Table 1:** Traditional uses of different parts of *FB* in different disorders.

Plant part	Traditional uses	References
Root	Diabetes, Respiratory disease, Skin disease, Gonorrhea, Vomiting	[15,20]
Bark	Asthma, Diabetes mellitus, Dysentery, Diarrhea, Leucorrhea, Menorrhagia, Toothache, Erysipelas, Hemorrhoids, Nervous Disorders, Laxatives, Skin disease, Ulcers, Mouth sores	[11,15,18]
Latex	Rheumatism, Inflammatory disease, Spermatorrhoea, Blood purification, Uro-genital disorders,	[9,19,20]
Leaf	Dysentery, Diarrhea, Hemorrhoids, Ulcer, Leucorrhoea, Lumbago, Sores, Ulcers Pains, Bruises	[20,23]
Aerial roots	Syphilis, Biliousness, Dysentery, Liver inflammation	[20]
Seed	Peptic ulcers	[9]
Miscellaneous	Blood disorders, Bone disorders, Endocrine disorders, Gastric disorders, Reproductive disorder, Urinary disorder, Fever, Biliousness, Ulcers, Erysipelas, Vomiting, Vaginal Complains, Inflammations, Leprosy, Abscess	[20,59]

## 4 Pharmacognosy

### 4.1 Habitat

*FB* belongs to the Moraceae family and grows well in tropical as well as semi-tropical regions. It is indigenous to Asian countries including India, Burma, China, Thailand, Malaysia, and other Southeast Asian countries.

### 4.2 Morphology

It is a giant evergreen tree standing up to 30 m high above the ground level with numerous aerial roots descending down the branches. The bark is smooth, thick, and green in colour in juvenile stage whereas it is greyish-white when mature. The fresh cut area of bark is pinkish in colour and secretes milky latex. Leaves are simple, with an alternate arrangement. They are leathery, ovate to elliptic, 4–6 inches long with reticular venation and a rounded or subcordate base. Unripe fruits are dark red achenes, 15 to 20 mm in diameter, globose, fleshy and fixed in axillary pairs and they turn dark purple when ripe. It has very small (∼18 mm) and distinct male, female, and imperfect female flowers with male ones packed adjacent to the receptacle mouth while female flowers have a shorter perianth and a long style.

## 5 Phytochemical constituents

Phytochemical investigations of *FB* show a variety of bioactive chemical constituents that are responsible for its broad pharmacological activities related to the prevention and treatment of thromboembolism, either directly or indirectly. It contains bioactive compounds from diverse classes including alkaloids, phenols, cardiac glycosides flavonoids, tannins, saponins, steroids, triterpenes, xanthoproteins, and coumarins (Table 2) [18].

**Table 2:** Phytochemical constituents of various parts of *FB*.

CLASS OF COMPOUND	PLANT PART	COMPONENTS	REFERENCE
**Phenolic compounds**	Roots	Lutein, Anthocyanin Cyanidin 3-Glucoside Equivalent (CGE), Chlorogenic Acid, Caffeic Acid, Quercetin, Naringenin, Kaempferol, Morin, Malondialdehyde	[21]
	Bark	Leucocyanidin-3-O-β-D-glucopyrancoside, Leucopelargonidin-3-O-β- glucopyranoside, Leucopelargonidin-3-O-α-L rhamnopyranoside,5,7-dimethylether-leucopelargonidin-3-0-alpha-L-rhamnoside	[9]
	Leaf	Rhein, Anthraquinone, Gallocatechin, Theaflavin-3, 3′-digallate, Flavone, Rutin, Quercetin-3-galactoside, leucodelphinidin, Gallocatechin, Kaempferol, Apigenin	[8,9,34]
**Flavonoid**	Leaf	Catechin, Genistein	[8]
	Fruit	Quercetin, Myricetin, Gallic acid, Caffeic acid, Chlorogenic Acid, Coumaric acid, Ferulic acid, Ellagic acid	[71,72]
**Terpenoids**	Aerial roots	Bengalensinone, Benganoic acid, Phytol, Globulol, Lanosterol, Lupeol, Amyrin Acetate, Lupenyl Acetate, Friedelanol, Cyclolaudenol, Epifriedelanol	[[8], [9], [10]]
	Bark	Lupeol, lupeol acetate, α-amyrin acetate, Gluanol acetate, Lanostadienylglucosyl cetoleate	[9]
	Leaf	Friedelin, β-sitosterol, lupeol, β-amyrin, 3- Friedelanol, Betulinic acid, 20-traxasten-3-ol), Taraxosterol	[8,9]
**Tannins**	Leaf	Galocatechin, Gallic acid	[9]
**Coumarin**	Leaf	Psoralen, Bergapten, Rhein	
**Miscellaneous**			
**Glycoside**	Bark	20-tetratriaconthene-2-one, 6-heptatriacontene-10-one, pentatriacontan-5-one, beta sitosterol-alpha-D-glucose	[60]
**Amino acid**	Fruit	Cysteine, Glutamine, Methionine, Tryptophan, Arginine, Methionine, Citrulline, Hydroxyproline, Glutathione	[8,73]
**Fatty acid**	Seed	vernolic acid (8.2%), malvalic acid (3.7%) and sterculic acid (1.6%) along with the other normal fatty acids like lauric acid (1.5%), myristic acid (1.3%), palmitic acid (35.2%), stearic acid (4.2%), oleic acid (20.3%), linoleic acid (15.4%) and linolenic acid (8.7%)	[45]
**Phytosterol**	Bark	Lanostadienylglucosyl cetoleate, Bengalensisteroic Acid acetate, α-Amyrin acetate	[8]
**Benzene**	Heart wood	Tiglic acid	[8]

### 5.1 Phenolic compounds

*FB* contains phenolic compounds including flavonoids such as Quercetin, Naringenin, Kaempferol, Quercetin-3-galactoside, etc. that have been isolated from its various parts [8,21]. Quantitative analysis revealed a significant amount of flavonoid content in the aqueous fraction (0.5148 ± 0.02 mg GAE/g dE) and hydroalcoholic extract (97mg/gm ± 5.10) of stem bark [18,22]. Quercetin-3-galactoside and rutin are found to be present in the leaves [23]. The biological and oxidative properties of flavonoids are responsible for their cardioprotective, anti-inflammatory, and anti-oxidative activities. There is ample acceptable evidence present that indicates the role of bioflavonoids, in free radical scavenging activity, enhancement of endothelial-derived nitric oxide activity as well as prevention of Low-Density Lipoprotein-Cholesterol (LDL C) oxidation [24], endothelial activation inhibition [25], and inhibition of platelet aggregation [26], and hence they probably reduce the risk of thrombosis.

A few handful of research have been conducted to elicit the antithrombotic characteristics of certain flavonoids which are found to be present in *FB* as well [27]. The anticoagulant and antithrombotic effect of quercetin and quercetin-3-O-β-D-glucoside (isoquercetin) was inferred through the inhibition of production of fibrin clots and blood clotting by impeding the enzymatic activity of FXa and thrombin and reducing epinephrine and collagen-induced platelet activation. Another flavonoid named Kaempferol showed anti-thrombotic action in collagen/epinephrine- and thrombin-induced acute thromboembolism models and FeCl_3_-induced carotid arterial thrombus models [28]. Furthermore, the antiatherogenic effects of leucopelargonin and leucocyanin were investigated in cholesterol rats and results showed a remarkable decrease in the atherogenic index, faecal excretion of bile acids, hepatic bile acid level, neutral sterols, lipogenic enzyme activities as well as HMG-CoA reductase in the liver significantly [13]. Hence, due to the presence of these flavonoids, *FB* may be clinically useful to prevent or treat thrombotic conditions.

### 5.2 Terpenoids

Different parts of *FB* have been noted to contain a wide range of terpenoids like lupeol, α-amyrin acetate, lupeol acetate, betulinic acid, gluanol acetate, friedelin, etc. [9,29]. GC-MS analysis of aerial roots from *FB* reported the presence of tri-terpenes namely phytol, globulol, lanosterol, lupeol, amyrin acetate, lupenyl acetate, friedelanol, cyclolaudenol, epifriedelanol [30]. However, the leaves of *FB* contain friedelin, 3-friedelanol, beta-sitosterol, 20-traxasten-3-ol, lupeol, or betulinic acid, and β-amyrin [9].

Lupeol identified and isolated from the dry leaves of *Elephantopus scaber* Linn. exhibited platelet aggregation activity at different concentrations [31]. Epifriedelanol and friedelin obtained from crude methanolic extract of leaves of *Cissus trifoliata* (L.) showed significant thrombolytic activity by *in-vitro* assay against distilled water and streptokinase (30,000 IU) as negative and positive thrombolytic controls respectively [32].

**Fig. 1:**
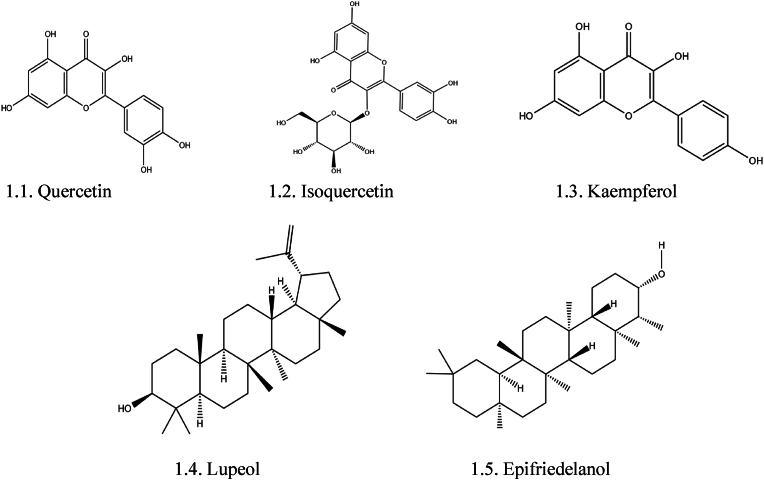
Structure of important flavonoids and terpenoids in *FB*: 1.1. Quercetin, 1.2. Isoquercetin, 1.3. Kaempferol, 1.4. Lupeol, 1.5. Epifriedelanol.

Few flavonoids and terpenoids isolated from *FB* are displayed in Fig. 1.

### 5.3 Tannins

Tannins in general exhibit anti-platelet activity, oxidative stress inhibition, maintain hemostasis and prevent nitric oxide (NO), prostacyclin (PGI2), and tissue-type plasminogen activator (t-PA) mediated disruption of cell membranes. Quantitative analysis showed a significant amount of tannin content in the micro-oven-dried fruit of *FB* (38.27 ± 06.03 mg GAE/g extract) [21]. *FB* bark also contains high levels of tannins [33]. Another study showed the presence of tannin in different solvents, while the highest was found in the aqueous fraction of the bark (3.787 ± 0.043 mg GAE/g dE) [18]. The methyl alcohol extract of leaves was reported to possess antioxidant capacity with an abundant quantity of tannins [34].

Catechin, epigallocatechin, and Gallic acid are the major tannins present in *FB*. Studies suggested that green tea catechins and epigallocatechin gallate extracted from green tea showed significant antithrombotic actions. It inhibited pulmonary thrombosis-related death in mice with a substantial increase in the bleeding time of mouse tail of conscious mice and suppressed *ex-vivo* rat platelet aggregation mediated by adenosine diphosphate and collagen in a dose-related manner. It also restrained ADP, collagen, epinephrine, and calcium ionophore A23187 induced human platelet aggregation *in-vitro*, in a dose-related manner [35]. Another study demonstrated the anti-platelet activity of green tea catechin due to inhibition of TXA2 formation by inhibiting arachidonic acid liberation and TXA2 synthase [36]. In addition to this, Gallic acid was found to suppress platelet aggregation, platelet-leukocyte aggregation, and P-selectin expression in a directly proportional manner to concentration. It further prevented the elevation of intracellular calcium and attenuated phosphorylation of PKCα/p38 MAPK and Akt/GSK3β on platelets stimulated by the stimulants ADP or U46619 [37]. These findings suggest a potential medicinal application of tannins thrombotic conditions.

### 5.4 Coumarins

**Fig. 2:**
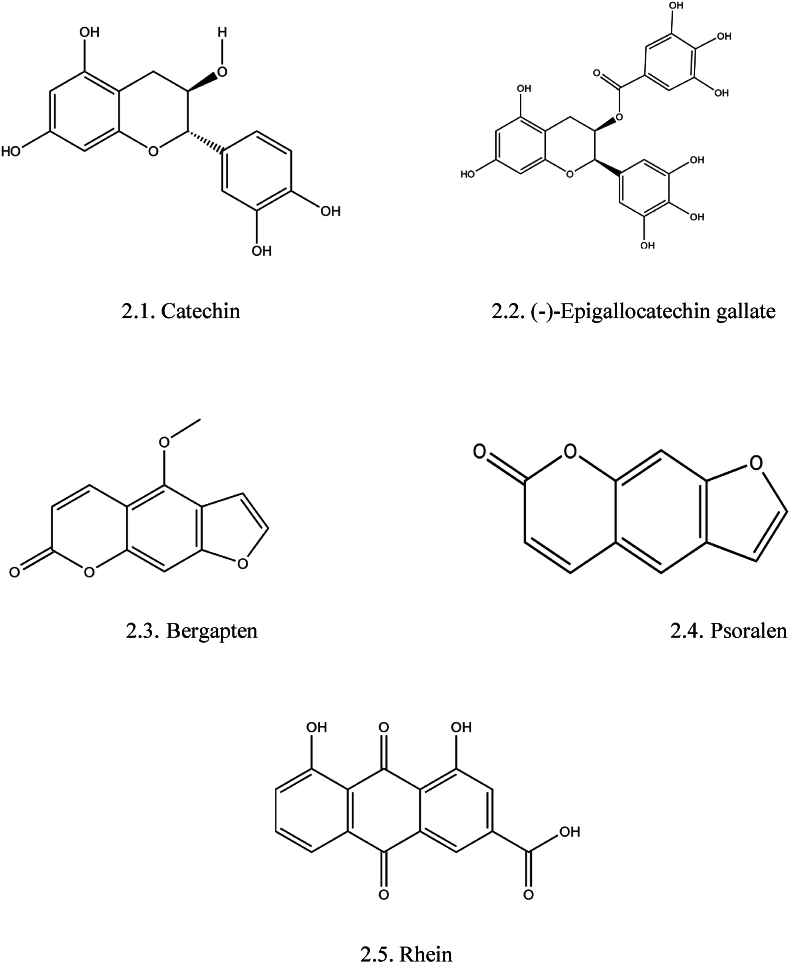
Structure of important tannins and Coumarins isolated from *FB*: 2.1 Catechin, 2.2 (−)-Epigallocatechin gallate, 2.3 Bergapten, 2.4. Psoralen, 2.5 Rhein.

The fruits, seeds, leaves, and bark of FB are a rich source of coumarins (Fig. 2) [9,38]. Bergapten (5-methoxypsoralen) and psoralen are the furocoumarin compounds that are abundantly found in the seeds of FB [39]. In addition to these, the leaves contain one furanocoumarin derivative, namely rhein [9]. Coumarins are competitive inhibitors of Vitamin K in the biosynthesis of prothrombin. Also, they exert anti-inflammatory, anticoagulant, antioxidant, and enzyme inhibition effects [40]. According to *in-vitro* assay results, bergapten and psoralen extracted from Angelica shikokiana, were shown to exhibit considerable antiplatelet action against ADP and arachidonic acid-induced platelet aggregations in a concentration-dependent manner [41].

### 5.5 Miscellaneous

#### 5.5.1 Glycosides

**Fig. 3:**
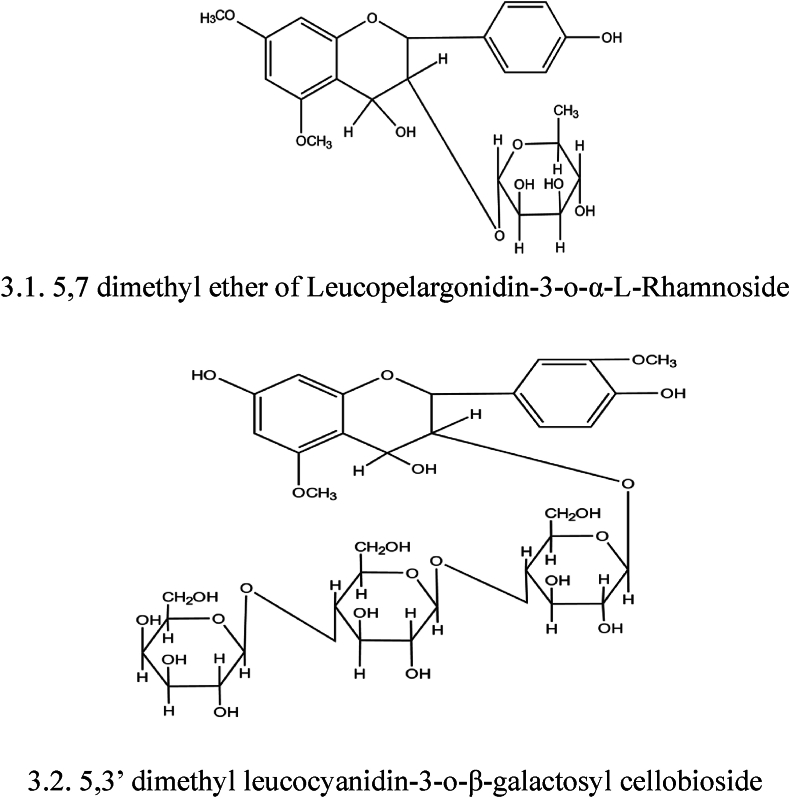
Structure of Glycoside isolated from *FB*: 3.1. 5,7 dimethyl ether of Leucopelargonidin-3-o-α-L-Rhamnoside, 3.2. 5,3′ dimethyl leucocyanidin-3-o-β-galactosyl cellobioside.

FB is one of the rich sources of glycosides like 20-tetratriaconthene-2-one, 6-heptatriacontene-10-one, pentatriacontan-5-one, and beta sitosterol-alpha-D-glucose. The crude methanolic extract of bark confirmed the presence of cardiac glycoside, while the methyl alcohol, hexane, and ethyl acetate extracts from the aerial roots showed the presence of both cardiac and steroidal glycosides [10,18]. Also, the presence of a fatty acid glycoside (2-O-a-L-rhamnopyranosyl-hexacosanoate-b-D-glucopyranosyl ester) was detected in the methanolic extract of leaves. That exhibited antioxidant activity and has potential for inhibition of acetylcholinesterase [42]. Glycoside of leucopelargonidin isolated from the bark of *FB* exhibited a remarkable *anti*-lipidemic effect via enhanced faecal excretion of sterols and bile acids in moderately diabetic rats (Fig. 3) [43].

#### 5.5.2 Amino acids

*FB* has been reported to have one of the highest sources of amino acid present in fruit protein [44]. Concentrated aqueous extract of the seeds and fresh fruits contained various amino acids such as cysteine, glutamine, methionine, tryptophan, arginine, citrulline, and hydroxyproline. Glutathione, an important antioxidant, has also been isolated from the fruit of *FB* [8].

#### 5.5.3 Fatty acid

Vernolic, malvalic, and sterculic acids have been found to be present in *FB* seed oil together with lauric, myristic, palmitic, stearic, oleic, and linolenic acids [8,45]. Linolenic acid is found to exhibit antithrombotic action by reducing P-selectin secretion, GP IIb/IIIa expression, and by reducing the expression level of PI3K and Akt [46].

#### 5.5.4 Phytosterol

Lanostadienylglucosyl cetoleate and bensisteroic acid ester were found to be present in methanolic extract of the stem bark of *FB* [8,45,47]. Evidence from various studies shows that phytosterols reduce atherosclerosis by means of both useful alterations in High Density Lipoprotein (HDL) and LDL metabolism and inflammatory pathways [48]. Phytosterols have also been noted to exhibit cholesterol-lowering effect, high LDL receptor expression, and lower circulation LDLC concentration [49].

Different plant parts and their distinguished extracts showed various components which are indicating the effect of *FB* towards the anti-thromboembolic effect as shown in Table 2.

## 6 Pharmacological studies

**Fig. 4:**
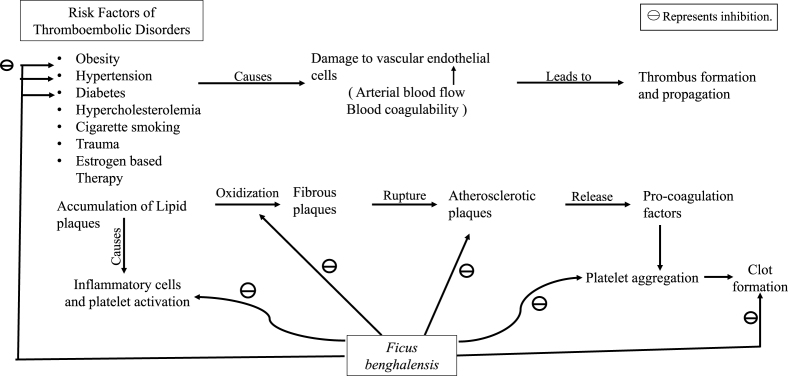
Pharmacological activities of *FB* in the management of thromboembolic disorders.

*FB* has been vastly studied for multiple pharmacological effects which are concerned with both prophylaxis and treatment of the thromboembolic disorder (Fig. 4).

### 6.1 Anticoagulant activity

The fractionated crude methanol extracts of leaves of *FB* using liquid-liquid partition with ethyl acetate*,* n-hexane, and chloroform in healthy human blood plasma, showed anticoagulant action, with delayed “prothrombin time (PT)” (21.7 ± 1.2 s) compared to the normal control (13.3 ± 0.6 s). The prothrombin values of n-hexane ranged from 17.3 ± 0.9 to 21.0 ± 1.0 s, chloroform, and ethyl acetate ranged between 17.7 ± 0.7 to 22.0 ± 1.1 and 17.8 ± 0.9 to 24.0 ± 1.5 s, respectively. It also showed significantly higher activated partial thromboplastin time (APTT) than normal and varied from 51.7 ± 2.4 to 72.3 ± 5.4, 49.7 ± 6.1 to 71.7 ± 5.5, and 47.7 ± 3.3 to 69.7 ± 2.9 s, respectively, for the n-hexane, chloroform, and ethyl acetate fractions [12]. This result clearly shows that *FB* has anti-coagulant properties, which may be supported by its notable phenolic and flavonoid content.

### 6.2 Platelet anti-aggregation activity

Water soluble fractions of ethanolic extracts of leaves of *FB* in concentrations of 8 mg/ml exhibited significant platelet *anti*-aggregatory activity against ADP-induced platelet aggregation in an *in-vitro* study. It inhibited platelet aggregation by 77.26 ± 0.7 % (8 mg/ml), and the IC50 value was obtained at 4.87 ± 0.38 mg/ml [14].

### 6.3 Antiatherogenic activity

Leucopelargonin and leucocyanin isolated from the bark of *FB,* along with quercetin in a dose of 100 mg/kg/day significantly decreased the liver-to-body weights (7–9%) in cholesterol-fed male SD rats. The activities of HMG-CoA reductase and lipogenic enzymes in the liver, lipoprotein lipase in the heart, adipose tissue, and plasma LCAT, as well as ester cholesterol in the liver, all declined markedly. Additionally, the hepatic level of bile acids increased, so as the faecal excretion of bile acids and neutral sterols, while the action of glucose-6-phosphate dehydrogenase decreased. The leucopelargonin derivative and quercetin showed a slightly better and more significant effect than the leucocyanin derivative [13].

### 6.4 Hypolipidemic activity

The aqueous extract of *FB* bark significantly showed a hypolipidemic effect in alloxan-induced diabetes mellitus in rabbits. Medication for one month at a dose of 50 mg/kg body weight/day reduced the levels of total cholesterol in serum from 82 ± 11 mg% and 118 ± 10.6 mg% to 42.7 ± 3.1 mg% and 51.7 ± 4.7 mg% in sub-diabetic and diabetic rabbits respectively. Low-Density Lipoprotein cholesterol and Very Low-Density Cholesterol values were found to reduce from 34 ± 10 mg% and 95 ± 24 mg% to 16 ± 3 mg% and 29 ± 4 mg respectively. Triacylglycerol (TAG) levels reduced from 121 ± 21.6 mg% and 416 ± 70 mg% to 45 ± 5 mg% and 81 ± 27.5 mg% respectively in sub-diabetic and diabetic rabbits [50]. The aerial roots of the plant showed a significant hypolipidemic and hepato-protective effect in STZ-induced diabetic rats with a significant increase in HDL cholesterol level and a decline in the levels of Total Cholesterol (TC), Triglycerides (TG), LDL, and Very Low-Density Lipoprotein (VLDL) [15]. Leucodelphinidin derivative (High Density Lipoprotein Cholesterol *i.e*.HDL-C) and quercetin derived from the bark showed a marked reduction in TC, LDL-C, atherogenic index, and an increase in the HDL-C levels in hyperlipidaemic rats by increasing faecal excretion of bile acids and cholesterol [51].

### 6.5 Hypotensive activity

In normotensive and angiotensin II-induced hypertension rats, an aqueous preparation of *FB* stem bark demonstrated considerable hypotensive action. Intravenous infusion of 10 mg/kg aqueous extract showed a decrease in systolic blood pressure (SBP), diastolic blood pressure (DBP), mean arterial blood pressure (MABP), and heart rate (HR) in normotensive rats compared to the control group by 10, 17, and 29 %, respectively while, in angiotensin II-induced hypertension group, *FB* extract successfully decreased the SBP, DBP, and MABP by 27, 30, and 29 %, respectively without an apparent decrease in HR [11].

### 6.6 Anti-inflammatory activity

Evidence from various studies suggests the strong anti-inflammatory action of *FB*. The oral dosing of ethanolic extracts of young plant bark exhibited a remarkable reduction in inflammation in the carrageenan-induced rat paw oedema model and cotton pellet granuloma model compared to indomethacin in a dose-dependent manner [21]. The aqueous extract of the stem bark showed a significant reduction in colon mucosa damage index, disease activity index, and myeloperoxidase in 2,4,6-trinitrobenzenesulfonic acid (TNBS) induced inflammatory bowel disease in albino Wistar rats. The Malondialdehyde (MDA), Nitric oxide (NO) levels, and mast cell degranulation decreased significantly while the Superoxide dismutase (SOD) activity increased in the colon. Also, histopathological analysis of the colon of treated rats showed less progression of inflammatory bowel disease (IBD) and was characterized by a decrease in hyperplasia and oedema, a decline in the infiltration of the inflammatory cells, mild necrosis, and ulceration [51]. The anti-inflammatory activity of the ethanolic extract of leaf was elicited in another study, with a significant reduction in paw volume (65.21%) at a dose of 200 mg/kg [17].

**Fig. 5:**
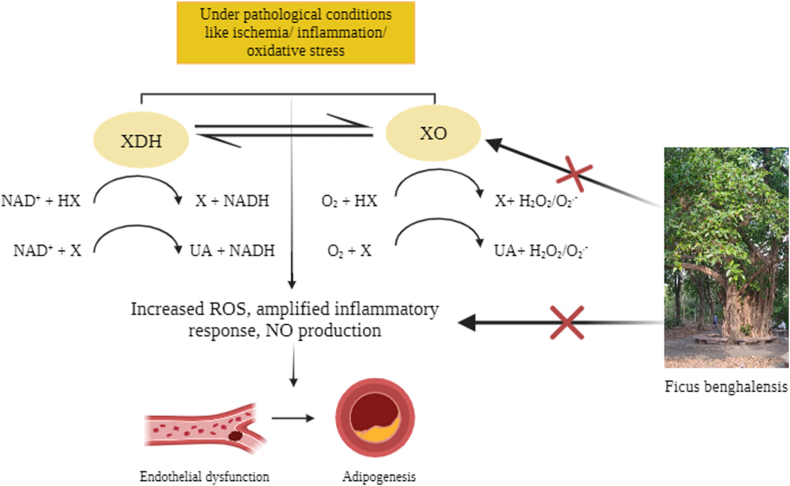
Anti-inflammatory activity of of *FB* bark.* ROS: Reactive Oxygen Species; NO: Nitric Oxide; XDH: Xanthine dehydrogenase; XO: Xanthine oxidase; NAD + : Oxidized form of nicotinamide adenine dinucleotide; NADH: reduced form of nicotinamide adenine dinucleotide; HX: Hypoxanthine, X: Xanthine; UA: Uric acid; O_2_ : Oxygen; H_2_O_2_ : Hydrogen peroxide; O_2_ - : superoxide. *Arrow with red cross indicates reduction/limiting.

Moreover, the *in-vitro* anti-inflammatory property of different extracts of the bark was also evaluated by various researchers by estimating the human red blood cell membrane stabilization (HRBC) method and the results showed significant stabilization towards membrane at a concentration of 200 mg/ml in comparison to the standard drug, diclofenac sodium [16,52]. Another study discovered that the fatty acid glycoside extracted from *FB* is a natural EGFR inhibitor, NO-releasing, and COX-inhibiting anti-inflammatory drug through suppression of the EGFR/Akt/PI3K pathway. It also reduced TNF-α, IL-6, and PGE2 expression [53]. Another study showed that the oven-dried fruits of *FB* showed high levels of anti-inflammatory action through protein denaturation assay (44.79 ± 3.26% at 0.5 mg sample) [33]. A gene expression study demonstrated strong anti-inflammatory activity of the hydroalcoholic extract of the bark by downregulation of TNF-α expression and upregulation of IL-10 expression through xanthine oxidase inhibition as shown in Fig. 5 [54].

Human xanthine oxidoreductase (XOR) performs major functions like purine catabolism that includes conversion of hypoxanthine to uric acid through xanthine dehydrogenase (XDH), production of ROS by xanthine oxidase (XO) activity and NADH oxidase activity and nitrite reductase activity that generates nitric oxide. XO delivers electrons directly to molecular oxygen (O_2_), thus generating the reactive oxygen species (ROS) i.e., superoxide anion (O^2−^), and hydrogen peroxide (H_2_O_2_). Further, hypoxia-mediated acidic pH and low O2 tension also increase the production of O^2−^ via decreased nitric oxide (NO) formation through NO synthase and increase its potential to uncouple. All free radicals thus formed including H_2_O_2_, O_2-_ and NO have an oxidizing effect, thereby contributing to oxidative stress.

In a pathological state, the circulating XOR gets converted to oxidase form and binds to endothelial cells inducing proinflammatory response (TNF) and organ damage. The proinflammatory activity by XOR-derived ROS affects the microvascular lining and induces atheromatous plaque formation (adipogenesis). Also, studies show that ROS facilitates adipocyte differentiation by accelerating mitotic clonal expansion. The pathological adipocyte-derived factors disrupt vascular homeostasis and contribute to endothelial vasodilatory dysfunction leading and subsequent hypertension. The pro-inflammatory cytokine TNF stimulates the production of endothelin 1 and angiotensin which leads to vasoconstriction and hypertension.

Sabi et al. in their invitro study demonstrated that *Ficus benghalensis* hydro-alcoholic bark extract showed dose-dependent reduction in xanthine oxidase activity, ROS, NO levels, and pro-inflammatory cytokine (TNF) and upregulation of anti-inflammatory cytokine (IL10) thus exhibiting strong anti-inflammatory activity [54]. Hence, this demonstrates the potential use of FB in inhibiting adipogenesis further endothelial dysfunction, and hypertension leading to following thromboembolic disorders.

### 6.7 Antioxidant activity

Strong anti-oxidant properties of alcoholic extracts of fruits, aerial root, leaves, bark etc. Were reported through 1-1-diphneyl-2-picrylhydrazyl (DPPH), nitric oxide, superoxide, and lipid peroxidation inhibition assays with IC50 values 8.16, 14.81, 25.66 and 16.32 μg/ml respectively [55]. In another study, 5,7-dimethyl ether of leucopelargonidin, 3-0-alpha-L rhamnoside, and 5,3′-dimethyl ether of leucocyanidin 3-0-alpha-D galactosyl cellobioside derived from the bark of *FB* showed significant antioxidant effects [13]. Another study demonstrated the protective effect of methanolic extract of aerial roots on isoniazid-rifampicin-induced hepatotoxicity, with significant reduction in the levels of serum ALP, ALT, and AST, while an increase in total protein and glutathione levels in a dose-dependent manner was also observed [56].

Among various *In-vitro* studies, the plant showed important activities responsible in the management of thromboembolic disorder (Table 3). The aqueous extract of stem bark of *FB* showed significant inhibition of microsomal lipid peroxidation (LPO) in a concentration dependant manner with IC50 value 80.24 μg/ml [57]. Methanolic extract of leaves showed strong anti-oxidant activity through a concentration-dependent increase in DPPH radical scavenging with an IC50 value 11.21 μl, a dose-dependent increase in total antioxidant activity, iron chelating activity as well as reducing power was also observed [34].

**Table 3:** Overview of *in vitro* studies related to *FB* in the management of thromboembolic disease.

S. No.	Effect	Plant parts	Solvent	Cell system (#) and *type of assay* (*)	IC50	Target	Phyto-constituent	Reference
1.	Anticoagulant	Leaves	Methanol	# Healthy human blood plasma cell * Prothrombin time (PT) * Activated partial thrombo-plastin time (APTT)	–	• Delayed PT and APTT	Phenol, Flavonoid	[12]
2.	Platelet anti-aggregation	Leaves	Ethanol	# Human blood samples -Platelet rich plasma (PRP) -Platelet poor plasma (PPP) * Platelet aggregation inhibition activity assay (channel aggregometer)	4.87 ± 0.38 mg/ml	• Inhibition of platelet aggregation	–	[14]
3.	Antioxidant	Leaves	Hydroalcoholic (Fraction: n-hexane, n-butanol, chloroform Water)	* Free radical scavenging activity using: −1, 1-diphenyl-2-picryl-hydryzyl −2, 2′-azino-bis (3-ethylbenzthiazoline-6-sulfonic acid) radicals	* DPPH Assay Hydroalcoholic: 32.3 ± 1.320 μg/ml n-Hexane: 28.2 ± 0.993 μg/ml n-Butanol: >1000 μg/ml Chloroform: >1000 μg/ml Water: 125.0 ± 0.025 μg/ml * ABTS Assay Hydroalcoholic: 52 ± 0.722 μg/ml n-Hexane: 58.2 ± 0.714 μg/ml n-Butanol: 491 ± 0.555 μg/ml Chloroform: >1000 μg/ml Water: 20.3 ± 0.133 μg/ml	• Increase in DPPH scavenging activity in concentration- dependant manner. • Increase in ABTS scavenging activity in concentration- dependant manner.	–	[59]
		Latex	Methanol	* DPPH radical scavenging activity * FeCl_3_ radical scavenging activity * Phosphor-molybdenum radical scavenging activity	* DPPH: 28.63 ± 0.16 μg/ml * FeCl_3_ radical: 49.82 ± 1.00 μg/ml * Phosphor-molybdenum radical: 31.84 ± 0.12 μg/ml	• Decrease in DPPH concentration • Decrease in concentration of FeCl_3_ radical. • Decrease in concentration of Phosphor-molybdenum radical	Glycoside, Alkaloid, Tannin, Phenol, Flavonoid	[60]
		Stem bark	Aqueous Butanol fraction	* Ex vivo inhibition of lipid peroxidation (LPO) * DPPH radical scavenging activity	80.24 μg/ml	• Inhibition of LPO • DPPH inhibition	Phenol, Flavonoid Tannins, Quercetin	[18,55,57]
		Aerial root	Methanol Ethanol Aqueous	* DPPH radical scavenging assay * FRAP Assay (ferric reducing antioxidant power)	–	• Increased DPPH scavenging action (Methanolic extract > Ethanol extract) • FRAP Assay: increased reducing ability of extract (Methanol extract > Ethanol extract) • Increased reducing ability	Phenols, Flavonoids	[30,74]
		Root	Petroleum ether Ethyl acetate Alcohol Water	* DPPH scavenging activity * Hdroxyl radical scavenging activity * Reducing capacity * Hydrogen peroxide activity	* DPPH: 93.22 μg/ml * Hydrogen peroxide scavenging activity: 0.65 mg mL^-1^	• Decrease in concentration of DPPH radical • Increased Hydrogen peroxide scavenging activity	–	[74]
4.	Anti- inflammatory	Bark	Methanol Ethanol Water Hydro-alcohol	* HRBC method	–	• Stabilization towards HRBC membrane	–	[52]

PT: Prothrombin Time; APTT: Activated Partial Thrombo-plastin Time; DPPH: 1, 1-diphenyl-2-picryl-hydryzyl; ABTS: 2, 2′-azino-bis (3-ethylbenzthiazoline-6-sulfonic acid) radicals; LPO: Lipid peroxidation; FRAP: Ferric Reducing Antioxidant Power; HRBC: Human Red Blood Cell.

In another study, the strong anti-oxidant action of *FB* was supported via DPPH (IC50- 73.99 ± 2.22 μg/ml), Hydrogen peroxide (H_2_O_2_) (IC50- 50.67 ± 1.77 μg/ml) and Nitric oxide (NO) scavenging assay (IC50- 69.02 ± 2.57 μg/ml). While the total antioxidant capacity (TAC) (IC50- 51.45 ± 1.23 μg/ml), CUPRAC (Cu2+ to Cu + reducing assay) (IC50- 55.51 ± 0.54 μg/ml), Metal chelating assay (IC50- 55.95 ± 0.92 μg/ml) and ABTS scavenging assay (IC50- 45.73 ± 1.17 μg/ml) of plant extract was also showed its beneficial role against various disease conditions [58].

Hydroalcoholic extract of leaves showed different DPPH and ABTS scavenging activity in a concentration-dependent manner. Significant DPPH scavenging activity was observed in the order of: vitamin C (11.5 ± 0.052 μg/ml) > quercetin (15.4 ± 0.120 μg/ml) > n-hexane (28.2 ± 0.993 μg/ml) > hydroalcoholic extract (32.3 ± 1.320 μg/ml) > water (125.0 ± 0.025 μg/ml) > chloroform (>1000 μg/ml) > n-butanol (>1000 μg/ml). While, the ABTS scavenging activity was noted in concentration-dependent manner with Vitamin C (6.40 ± 0.015 μg/ml) > Quercetin (7.05 ± 0.115 μg/ml) > hydroalcoholic extract (52 ± 0.722 μg/ml) > n-hexane (58.2 ± 0.714 μg/ml) > water (20.3 ± 0.133 μg/ml) > n-butanol (491 ± 0.555 μg/ml) > chloroform (>1000 μg/ml) [52,59].

The *in vitro* antioxidant activity of methanolic extract of the latex also showed high DPPH scavenging activity along with IC50 value 28.63 ± 0.16 μg/ml, high ferric chloride scavenging with (IC50 49.82 ± 1.00 μg/ml) and a significant decrease in the concentration of phospho-molybdenum radical due to scavenging potential IC50 value 31.84 ± 0.12 μg/ml [60,61].

**Fig. 6:**
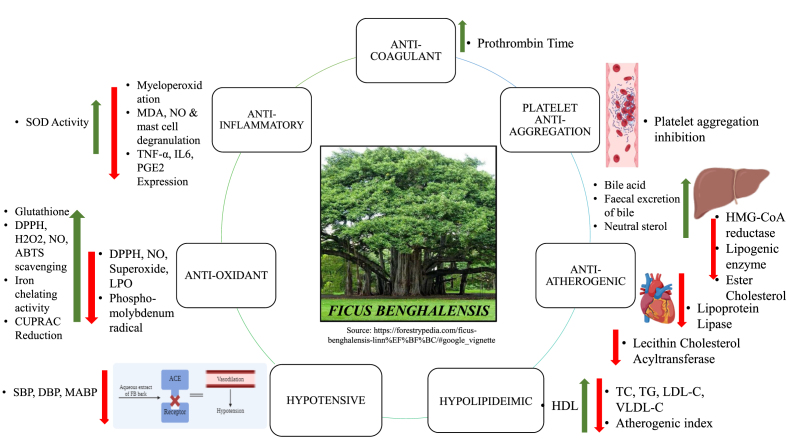
Mechanism of *FB* extracts towards prevention of thromboembolic disorders. * SOD: Superoxide dismutase; MDA: Malondialdehyde; NO: Nitric oxide; TNF-α: Tumour Necrosis Factor alpha; IL6: Interleukin 6; PGE_2_: Prostaglandin E 2; DPPH: 1, 1-diphenyl-2-picryl-hydryzyl; H_2_O_2_: Hydrogen Peroxide; ABTS: 2, 2′-azino-bis (3-ethylbenzthiazoline-6-sulfonic acid) radicals; CUPRAC: Cupric reducing anti-oxidant capacity; LPO: Lipid peroxidase; TC: Total cholesterol; TG: Tri glycerol; LDL-C: Low Density Lipoprotein Cholesterol; HDL-C: High Density Lipoprotein Cholesterol; VLDL-C: Very Low Density Lipoprotein Cholesterol; SBP: Systolic Blood Pressure; DBP: Diastolic Blood Pressure; MABP: Mean Arterial Blood Pressure; HMG-CoA: Hydroxymethylglutaryl-CoA. *Red arrow indicates decrease and Green arrow indicates increase

In addition to this, collective information of *FB* extracts and its overall biological activities are represented in Fig. 6, while detailed *in-vivo* studies is mentioned in Table 4.

**Table 4:** Overview of *in vivo* studies related to *FB* in management of thromboembolic disease.

S.No.	Effect	Solvent	Animal	Dose	Duration	Target	Phytoconstituent	Reference
1.	Anti-oxidative	Methanol	Rat	10, 50, 100, 500 μg	21 days	• Decrease in TBARS level and Increase in GSH level	Flavonoid, Terpenoid	[56]
		Aqueous	Rabbit	50 mg/kg/day	4 weeks	• Increase in SOD, CAT, glutathione peroxidase, glutathione reductase	–	[50]
		Aqueous	Rats	200 & 500 mg/kg	21 days	• Decrease in MDA, NO and Increase in SOD activity		[51]
		Ethanol	Rats	100, 200 & 400 mg/kg	–	• Decrease in SOD, LPO and CAT	Phenol	[57]
2.	Hypolipidemic	Aqueous	Rabbit	50 mg/kg/day	4 weeks	• Decrease in TC, TG, LDL-C, VLDL-C	–	[50]
		Aqueous	Rats	300 mg/kg	21 days	• Decrease in TC, TG, LDL, VLDL and Increase in HDL-C	–	[15]
		Suspension in Normal Saline	Rats	100 mg/kg/day	90 days	• Decrease in TC, Atherogenic index, LDL-C and Increase in HDL-C		[13]
		Alcohol	Rats	100 mg/kg/day	90 days	• Decrease in TC, Atherogenic index, LDL-C • Increase in HDL-C, total bile acid (in liver), faecal excretion of bile acid, neutral sterols	Leucodelphinidin, Quercitin	[75]
		–	Rats	–	4 weeks, 40 days	• Hypo-cholesteroleimic effect and enhanced faecal excretion of sterols and bile acids	–	[43,76]
3.	Anti-inflammatory	Methanol	Rats	Carrageenan-induced paw oedema @ 100, 200, 300, 400, 500 mg/kg	1-h prior injection	• Decrease in oedema	Flavonoid, Tannin	[19]
				1. Cotton pellet induced granuloma- 200, 400 mg/kg	8 days	• Inhibition of exudatory and granulatory phases.	Flavonoid, Tannin	
				2. Acetic acid-induced vascular permeability-200, 400 mg/kg	1-h prior injection	• Inhibition of increased leakage of dye into peritoneal fluid	Flavonoid, Tannin	
		Ethanol	Rats	300 & 600 mg/kg/day	7 days	• Decrease in paw volume	Flavonoid	[23]
		Aqueous	Rats	200 & 500 mg/kg	21 days	• Decrease in CMDI, DAI, MPO activities	Flavonoid, Terpenoid	[51]
4.	Anti-hypertensive	Aqueous	Rats	10 mg/kg	Single time administration	• Decrease in SBP, DBP, MABP, HR	Phenols/Flavonoid	[11]

TBARS: Thiobarbituric acid reactive substance; GSH: Glutathione; SOD: Superoxide dismutase; MDA: Malondialdehyde; NO: Nitric oxide; LPO: Lipid peroxidase; CAT: Catalase; TC: Total cholesterol; TG: Tri glycerol; LDL-C: Low Density Lipoprotein Cholesterol; HDL-C: High Density Lipoprotein Cholesterol; VLDL-C: Very Low Density Lipoprotein Cholesterol; CDMI: Colon Mucosa Damage Index; DAI: Disease Activity Index; MPO: Myeloperoxidase; SBP: Systolic Blood Pressure; DBP: Diastolic Blood Pressure; MABP: Mean Arterial Blood Pressure; HR: Heart Rate.

## 7 Toxicity studies

Plants may have compounds that have agonistic or antagonistic natures which can exhibit potential toxic effects, trigger hypersensitivity reactions, or sometimes lead to anaphylactic shock. As a result, it is critical to assess the unfavourable and toxic effects of isolated plant extracts and phytochemical substances intended for human treatment. Evidences from various studies highlight the safe dose and potential toxic effects of various parts of *FB*.

### 7.1 Aerial roots

The acute oral toxicity study of aqueous extract on healthy albino Wistar rats, showed normal rat behaviours and no mortality or toxic effects when orally administered with a single dose of 300 mg/kg aqueous extract 10 and 15 times [15]. Methanolic extract of *FB* aerial roots using Swiss albino mice at doses ranging from 500 to 5000 mg/kg at various dose levels showed no mortality or any signs of behavioural changes up to 48 h of treatment [56]. Similarly, another study showed that the ethyl acetate extracts of *FB* aerial roots in the dose of 5000 mg/kg body wt. did not induce any harmful effects on behaviour, neuro-motor abilities, body weight, water-feed intake pattern, liver and kidney functions or mortality in female Wistar rats throughout the 14-day trial in comparison to the corresponding control group. Thus, it can be inferred that the potential oral fatal dose of Ethyl Acetate Extracts of *FB* Aerial Roots is greater than 5000 mg/kg body wt. Which makes it safe for oral consumption [62]. Another acute toxicity study carried out on the ethanol and aqueous extracts of aerial roots at a dose of 3000 mg/kg body weight showed no mortality or negative changes in behavioural, neurological and autonomic profiles in the rats after 24 h and 72 h [63]. Successive methanolic extracts of aerial roots administered as a single bolus dose of 2000 mg/kg orally showed no mortality and physical/behavioural changes on rats when observed over 14 days [64].

### 7.2 Bark

In an acute toxicity study of oral administration of a partially purified preparation from the water extract of the bark showed LD_50_ value −1 mg/kg which was much higher and safer when compared to the control drug chlorpropamide (LD_50_ 760 mg/kg) [65]. Results from another study elicited that oral administration of 5000 mg/kg aqueous extracts of bark in Swiss Albino male mice showed neither treatment related signs of acute toxicity nor mortality during the course of 14 days treatment. Also, the same were observed in sub chronic 28 days oral toxicity study at high dose of 1000 mg/kg/wt [66]. In another study, ethanolic and aqueous extracts of the bark showed no signs of toxicity or mortality at 2000 mg/kg dose level at a total of 14 days [67].

High LD50 values and non-toxic effects were observed in male Wistar rats on a number of parameters including blood glucose, serum cholesterol, liver and kidney function tests, haemoglobin, total and differential leukocytes, and histopathological parameters of the liver, heart, and kidneys at high doses (50, 100, and 150 mg/kg) during the course of three months of chronic toxicity studies [65].

### 7.3 Fruit

Methanolic extract of *FB* fruit was studied for acute toxicity at dose of 2000 mg/kg i.p. in female albino mice. The extract did not cause any mortality even at repeated dosing using 3 new mice. Hence, 5000 mg/kg was taken as Lethal Dose (LD50) cut-off value as per fixed dose method of OECD guideline number 423 [68].

### 7.4 Seeds

Following a 14-day period, oral administration of a single dosage (2000 mg/kg body weight) of seed extracts to male and female Wistar albino rats did not result in any atypical clinical symptoms or behavioural patterns or evidence of acute toxicity [69].

### 7.5 Whole plant

On 14 days of therapy, the methanolic extract of the whole plant, given orally to female mice at dosages of 2000 and 5000 mg/kg bd.wt., exhibited no evidence of toxicity, confirming its safety against acute toxicity models [70].

Hence, to the best of our knowledge no such studies have been reported so far that highlights the potential toxic effects of *FB* extracts which makes it practically safe, non-toxic and well tolerated for long term use in managing thromboembolic disorders.

## 8 Conclusion and future perspective

*FB* has garnered much attention in the past as well as in the present due to its variety of medicinal uses owing to an array of biochemical constituents present in it. Findings from several experimental studies have indicated that *FB* may be effective against various thromboembolic disorders (Table 3, Table 4). Since thromboembolic diseases are multifactorial, the pleiotropic and multimode actions of *FB* have been indicated to prevent oxidative stress, inflammation, atherosclerosis, thrombosis, dyslipidaemia, and hypertension, proposing its effect. Although *FB's* antioxidant activity could be the primary reason for its protective effects, research suggests that phenolic compounds, that comprise a phenolic ring with one or more hydroxyl groups, are mainly accountable for scavenging free radicals, preventing lipid peroxidation, chelating metal ions, reducing atherogenic index and correcting dyslipidaemia. Moreover, the hydroxyl groups may control the generation ROS, inflammatory cytokines, blood pressure reduction, platelet activation, and damage to vascular endothelial cells. The data from current studies demonstrate the potential role of *FB* in cardiovascular health pertaining to thromboembolic disorders. However, further analyses should be carried out to assess the unexplored cardiac activities such as cardiotonic effects, diuretic/decongestive effects, anti-ischemic effects on endothelial functions, anti-ischemia-reperfusion injury, and anti-hypertrophy to measure the compound's perspective and rational use of the desired phytoconstituents. Since, it is not yet introduced for use as a medicinal agent, clinical trials should be done to confirm the safety and efficacy in patients with thromboembolic disease.

Besides that, the accuracy, reproducibility, and commercial viability of bioactive compound isolated from *FB* should be confirmed. In conclusion, the present review provides potential information on the studies that have already been carried out and close any research gaps for pharmacological aspects that might need value addition through some experimental studies to the data related to this species.

## Sources of funding

This research did not receive any specific grant from funding agencies in the public, commercial, or not-for-profit sectors.

## Declaration of Generative AI in Scientific Writing

None.

## Author contribution

AKS and DD conceptualized the idea, designed the review collected the data and prepared the first draft. VKV edited the draft and finalised the manuscript. VP collected the data from varied sources and compiled it. DSA and JB supervised and guided the manuscript writing. All the authors approved the final manuscript.

## Conflict of interest

The authors declare that they have no known competing financial interests or personal relationships that could have appeared to influence the work reported in this paper.
